# Development and validation of dynamic bioenergetic model for intermittent ergometer cycling

**DOI:** 10.1007/s00421-023-05256-7

**Published:** 2023-06-27

**Authors:** Julius Lidar, Mats Ainegren, David Sundström

**Affiliations:** Department of Engineering, Mathematics and Science Education, Sports Tech Research Centre, Mid Sweden University, Östersund, Sweden

**Keywords:** Cycle ergometer, Parameter estimation, Oxygen deficit, Oxygen kinetics, Anaerobic work capacity

## Abstract

**Purpose:**

The aim of this study was to develop and validate a bioenergetic model describing the dynamic behavior of the alactic, lactic, and aerobic metabolic energy supply systems as well as different sources of the total metabolic energy demand.

**Methods:**

The bioenergetic supply model consisted of terms for the alactic, lactic, and aerobic system metabolic rates while the demand model consisted of terms for the corresponding metabolic rates of principal cycling work, pulmonary ventilation, and accumulated metabolites. The bioenergetic model was formulated as a system of differential equations and model parameters were estimated by a non-linear grey-box approach, utilizing power output and aerobic metabolic rate (MR_ae_) data from fourteen cyclists performing an experimental trial (P2) on a cycle ergometer. Validity was assessed by comparing model simulation and measurements on a similar follow-up experimental trial (P3).

**Results:**

The root mean square error between modelled and measured MR_ae_ was 61.9 ± 7.9 W and 79.2 ± 30.5 W for P2 and P3, respectively. The corresponding mean absolute percentage error was 8.6 ± 1.5% and 10.6 ± 3.3% for P2 and P3, respectively.

**Conclusion:**

The validation of the model showed excellent overall agreement between measured and modeled MR_ae_ during intermittent cycling by well-trained male cyclist. However, the standard deviation was 38.5% of the average root mean square error for P3, indicating not as good reliability.

## Introduction

The skeletal muscles responsible for human locomotion are dependent on energy released from adenosine triphosphate, which can be replenished by the anaerobic alactic and lactic systems and the aerobic system, respectively. Due to the different rates and capacities of these systems, the alactic system is the main source of adenosine triphosphate during short sprints, whereas the lactic system is most prominent during longer sprints, and the aerobic system is the main contributor during prolonged exertion. Despite this general pattern, research shows that the anaerobic system is also important in endurance sports characterized by varying intensity, such as cycling and cross-country skiing (Skiba et al. 2012; Gløersen et al. 2020). Over varying terrain, the anaerobic system is utilized during periods of greater exertion, such as uphill sections, where the aerobic system cannot meet the metabolic demand, resulting in oxygen deficits that are repaid during periods of lower exertion (Gløersen et al. 2020). The anaerobic system is also used as a buffer during the ramp-up of the aerobic system, which has a much slower response. The alactic system is limited to short contributions due to the limited amount of available phosphocreatine, but it can also be rapidly recovered during periods of lower intensity to be utilized again (McCann et al. 1995). In theory, the glycogen stored in skeletal muscle cells could fuel the lactic system for a considerable amount of time. However, during high intensity exercise glycolysis causes metabolites to accumulate, which can disturb the homeostasis of the muscles over time, ultimately limiting further glycolysis and therefore greatly reducing the work capacity of the muscles (Stanley and Connett 1991).

Numerous attempts to mathematically describe the behavior of the human energy supply systems have been made. Morton and Billat (2004) presented a model applicable to intermittent exercise, which was further developed by Skiba et al. (2012, 2015). These models allow a finite energy store (comparable to anaerobic capacity) to be utilized and recovered, with the utilization and recovery rates dependent on metabolic demand. However, the models make no distinction between energy generated by the lactic and alactic systems, nor do they factor in the dynamic behavior of the aerobic system. Moreover, according to these models, energy from the aerobic system is provided at the critical power level at all times.

Regarding the dynamic behavior of the aerobic system, it is widely accepted it can be described as the sum of a constant baseline and three exponential functions with their own separate error signals, time constants, and time delays (Poole and Jones 2012). The first exponential function (the cardio dynamic component) is of small magnitude and duration and is therefore often incorporated into the second exponential function (the primary component). For the third exponential function (the slow component), several underlying mechanisms have been suggested, e.g., a drift in metabolic demand, reduced efficiency in recruited muscle fibers, and successive recruitment of less efficient muscle fibers (Poole and Jones 2012).

Several mathematical models that incorporate the described dynamic behavior of the aerobic system have been suggested. A model proposed by Artiga Gonzalez et al. (2019) gives a mean absolute percentage error of 2.1% when fitted to a race-like road cycling exercise, but this increases to 10.8% when the model was fitted to another semi-race-like protocol and then applied to the race-like exercise. Furthermore, the model relies wholly on power output measured by an ergometer, and as such any deviation from a purely linear relationship between power output and oxygen uptake is captured only by adjustments to oxygen dynamics modeling. In reality, purely linear demand is unlikely. For instance, there is likely to be additional metabolic demand related to the acidification of the muscles (Gaesser and Brooks 1984). As a result, the model proposed by Artiga Gonzalez et al. (2019) gives no further insight into the underlying control mechanisms of oxygen uptake, nor can it provide estimates of anaerobic metabolic rates. Another model proposed by Stirling et al. (2005) offers the possibility of calculating both oxygen demand and oxygen deficit (e.g., anaerobic contribution) but no way of separating lactic and alactic contributions. In terms of oxygen uptake response and underlying oxygen demand, this curve-fitting model provides no additional insights into the physiological mechanisms of oxygen uptake dynamics. Moxnes et al. (2014) tested this model for several constant power outputs with subsequent recovery and showed poor results at higher exercise intensities. The study also tested three additional models describing the dynamics of the aerobic system. The most complex model agreed well with measured aerobic metabolic rate although it did not capture the exact dynamics. However, the model used the lactic metabolic rate as input, approximated from measured blood lactate. This is a crude estimation and since lactic metabolic rate is used as input, the model offers no additional information on the dynamics of the lactic system nor the alactic system.

Several attempts to collectively describe the dynamics of all three energy supply systems and their interactions mathematically have been made based on the hydraulic tank approach proposed by Margaria (1976). In his model, the available energy from each of the energy supply systems is depicted as fluid in a tank and the utilization of energy as fluid flow from each of the tanks. The relative size and positioning of the tanks and the flow capacity of their interconnecting pipes define the dynamic behavior of the system. Morton (1986, 1990) further generalized this approach and derived restrictions based on empirical data. Sundström (2016) further developed the hydraulic tank approach with separate supplies of fat and carbohydrates and found it to show good agreement regarding relative aerobic contribution but overestimations of times to exhaustion. Lidar et al. (2021) calibrated Morton’s model, and a similar model with a combined anaerobic system, using individual data from simulated sprint cross-country skiing races and achieved good overall agreement. However, neither of the models were able to fully capture the dynamic behavior of the aerobic system. Moreover, the protocols used did not include any periods of significant recovery. Had this been the case, anaerobic recovery would likely have been exaggerated by the model. Firstly, because all excess aerobic energy, in relation to metabolic demand, is added to the anaerobic stores, while in reality, recovery efficiency of 100% is highly unlikely. Secondly, there is likely to be residual metabolic demand during passive (and possibly active) recovery following heavy exertion (Gaesser and Brooks 1984) which was not included in the speed-dependent linear relationship for metabolic demand used by Lidar et al. (2021).

Thus, to better understand metabolic processes during variable-intensity endurance exercise and predict human performance in endurance exercise, a robust mathematical model is needed—one that describes the interaction between metabolic energy demands and the three metabolic energy supply systems. Therefore, the aims of this study were (1) to develop a bioenergetic model describing both metabolic energy demands and the aerobic and anaerobic lactic and alactic metabolic rates from readily measurable quantities during intermittent cycling and (2) to assess the validity and reliability of the developed model based on individual aerobic metabolic rate.

## Method

A novel bioenergetic supply and demand model was developed from the bottom up. To assess the validity and reliability of the model, athletes performed cycle ergometer exercises while following purposive variable-power protocols. One protocol was used to individually adapt the bioenergetic model using non-linear grey-box parameter estimation. The individualized models were then applied to data from another similar protocol.

### Participants

Fourteen well-trained male cyclists (age: 35 ± 8 years, height: 181 ± 5 cm, weight: 74 ± 6 kg, $\dot{V}{\text{O}}_{{\text{2,peak}}}$: 66.2 ± 5.8 ml·min^−1^·kg^−1^) received written information and gave written consent to participate in the study, which was approved by the Swedish Ethical Review Authority (Dnr 2021-04147).

### Experimental protocols

Each subject completed power-controlled tests on three separate days on a cycle ergometer with 2–7 days in between test days (there were 13 days between test day 2 and 3 for one subject due to illness). The first day comprised a submaximal incremental protocol (P1a) and a maximal incremental protocol (P1b). The second and third days each consisted of an intermittent protocol (P2 and P3, respectively) with individualized constant ergometer power outputs. All subjects could view their power output, heartrate, and cadence on the ergometer monitor during the tests and were instructed to maintain a cadence of 90 rpm throughout all protocols.

#### Incremental protocols and individual power output calculations

Power output in P1a started at 80 W and increased by 20 W every 3 min until monitored ventilatory data indicated that a respiratory quotient (RQ) above 1.0 had been reached. A venous lactate sample was taken 30 s before each power output increment. Participants rested for about five minutes between P1a and P1b. Power output in P1b started at 100 W and increased 40 W every minute until volitional exhaustion (subject stopped cycling or cadence dropped below 70 rpm for more than 3 s). Participants were verbally encouraged during the last few minutes of the protocol.

From P1a, the venous blood lactate concentration ([bLa^−^]) vs power output was fitted to a third degree polynomial. A blood lactate baseline was established by calculating the mean of the first two values of [bLa^−^], then adding one additional value at a time to the mean calculation until the difference between the mean value and the next [bLa^−^] value exceeded 0.2 mmol L^−1^. This mean value was used as the baseline for [bLa^−^] and the lactate threshold (LT1) was the value 0.2 mmol·L^−1^ above the baseline. This [bLa^−^] difference of 0.2 mmol·L^−1^ was the rounded-up value of the typical error (0.12 mmol·L^−1^), determined with a selection of duplicate samples (*n* = 40) from the beginning and end of the study. The power output at LT1 (P_LT1_) and onset of blood lactate (P_OBLA_) were taken from the third degree polynomial at the LT1 [bLa^−^] and [bLa^−^] = 4 mmol·L^−1^, respectively.

Peak oxygen uptake ($\dot{V}{\text{O}}_{{\text{2,peak}}}$) for the individual power output calculations was calculated as the maximal 30 s mean during P1b. Spirometry data from the submaximal test was used to calculate MR_ae_ according to

1 $${\mathrm{MR}}_{\mathrm{ae}}=\left(1.232\cdot \mathrm{RQ}+3.8149\right)\cdot \dot{V}{\mathrm{O}}_{2}\cdot \frac{4184}{60}$$

where RQ is the nonprotein respiratory quotient (McArdle et al. 2009), $\dot{V}{\text{O}}_{{2}}$ is the oxygen uptake in L·min^−1^, and $\frac{4184}{60}$ is the transformation from kCal·min^−1^ to W. A linear regression of $\dot{V}{\text{O}}_{{2}}$ vs MR_ae_ for the submaximal power outputs in P1a (RQ ≤ 1) was extrapolated to $\dot{V}{\text{O}}_{{\text{2,peak}}}$ in P1b and used to extract the corresponding power output (P_VO2,peak_). Finally, the maximum power output (P_max_) was calculated as the power output at exhaustion during P1b minus 40 W times the fraction of the minute left until the next power increment (e.g., exhaustion with 15 s cycling left at 320 W would give ${P}_{\mathrm{max}}=320-40\frac{15}{60}=310$ W).

The individual values of P_LT1_, P_OBLA_, P_VO2,peak_, and P_max_ were used to decide the individual power output levels for P2 and P3.

#### Intermittent protocols

Seven power output levels for P2 and P3 were purposively chosen to engage the three metabolic energy supply systems to various extents. The power output levels are shown in Table 1.

**Table 1:** Ergometer power output levels used in the intermittent protocols (P2 and P3)

Level	Power output
L4.2	P_VO2,peak_ + 50% (P_max_ – P_VO2,peak_)
L4.3	P_VO2,peak_ + 25% (P_max_ – P_VO2,peak_)
L3	P_OBLA_ + 60% (P_VO2,peak_ – P_OBLA_)
L2	P_LT1_ + 60% (P_OBLA_ – P_LT1_)
L1	85% P_LT1_
Active rest (AR)	50% P_LT1_
Passive rest (PR)	0 W

**Fig. 1:**
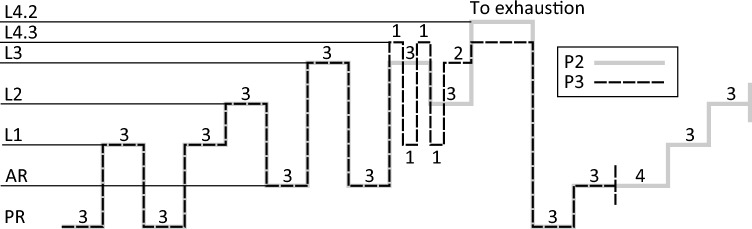
Intermittent test protocols used during test day 2 (P2) and test day 3 (P3) where the numbers reflect the duration in minutes of each period of constant power. The constant power levels are shown to the left where PR is passive rest (no pedaling), AR is active rest, and L1, L2, etc., are individual ergometer power levels (see Table 1) intentionally designed to activate the subjects’ different metabolic energy supply systems to varying degrees

The designs of P2 and P3 are shown in Fig. 1. All exercise and rest periods in the protocols were of specific, predefined durations, except the last periods of L4.2 and L4.3, respectively, which were continued until volitional exhaustion (subject stopped cycling or cadence dropped below 70 rpm for more than 3 s). Participants were verbally encouraged during the last few minutes prior to exhaustion. Participants were allowed to remove the spirometry mouthpiece for a short time (< 30 s) during active rest at two specified instances during each of the intermittent tests but were otherwise instructed to keep the mouthpiece in throughout the test (also at the point of exhaustion). Additionally, two subjects removed the mouthpiece for < 20 s at exhaustion.

### Bioenergetic model description

The developed bioenergetic model mathematically describes the behavior of the alactic, lactic, and aerobic energy supply systems. To enable this, it also models the underlying energy demand rates and a representation of active muscle lactate concentration ([mLa^−^]). The lactic system is the part of the glycolysis that causes an accumulation of lactate from the pyruvate that is not oxidized in the aerobic system. The model is given by Eqs. (2)–(13) as a state-space model where *u* denotes input (independent variables), *x* denotes state (mediating variables), and *y* denotes output (dependent variable). The bioenergetic model must adhere to the principle of energy conservation described as

2 $${\mathrm{MR}}_{\mathrm{dem}}={\mathrm{MR}}_{\mathrm{sup}}$$

where MR_dem_ is the metabolic demand rate and MR_sup_ is the metabolic supply rate.

#### Energy supply

The energy supply of the bioenergetic model can be expressed, in terms of metabolic rate, as

3 $${\mathrm{MR}}_{\mathrm{sup}}={\mathrm{MR}}_{\mathrm{al}}+{\mathrm{MR}}_{\mathrm{la}}+{\mathrm{MR}}_{\mathrm{ae}}={\dot{x}}_{1}+{x}_{2}+{x}_{3}+{\mathrm{MR}}_{\mathrm{rest}}$$

where MR_al_ = $\dot{x}_{1}$ is the alactic system metabolic rate, MR_la_ = *x*_2_ is the lactic system metabolic rate, and

4 $$y={\mathrm{MR}}_{\mathrm{ae}}={\mathrm{MR}}_{\mathrm{rest}}+{x}_{3}$$

is the aerobic system metabolic rate. MR_rest_ is both the metabolic demand and the aerobic metabolic rate at sitting rest, but without the metabolic demand due to ventilation, which is treated separately. *x*_*3*_ is the primary component of aerobic metabolic rate. The bioenergetic system metabolic rates ($\dot{x}_{1}$, *x*_2_, *x*_3_) and [mLa^−^] are governed by the following system of time-dependent differential equations:

5 $$\left\{\begin{array}{l}{\dot{x}}_{1}=(\text{M{R}}_{\rm dem}-\text{M{R}}_{\rm rest}-{x}_{3}-{x}_{2})/{E}_{\rm al,max}\\ {\dot{x}}_{2}=(\text{M{R}}_{\rm dem}-\text{M{R}}_{\rm rest}-{x}_{3}-{x}_{2})/{\tau }_{\rm la}-K\cdot {\dot{x}}_{3}\\ {\dot{x}}_{3}=(\text{M{R}}_{\rm dem}-\text{M{R}}_{\rm rest}-{x}_{3})/{\tau }_{\rm ae}\\ {\dot{x}}_{4}=({x}_{2}-{A}_{\rm red}\cdot {Z}_{\rm dem}\cdot {x}_{4})/{V}_{\rm m}\end{array}\right.$$

The rate of change of the aerobic metabolic rate $\dot{x}_{3}$ is controlled by an error signal MR_dem_* − *MR_rest_* − x*_3_, and a time constant τ_ae_ determining the response time. The error signal is the difference between the total metabolic demand rate MR_dem_ and the current aerobic metabolic rate MR_rest_ + *x*_3._ In a dynamic model formulation, this is equivalent to a mono-exponential response without time delay. We hypothesize that this mono-exponential in combination with drifting metabolic demand and high metabolic demands will capture the complete dynamic behavior of the aerobic system.

To our knowledge, the only proposed models of the lactic system are those within the hydraulic tank models that include a separate lactic system. Given the close relation between the lactic and aerobic systems, we propose that the rate of change of the lactic system *ẋ*_2_ is similarly regulated by an error signal (MR_dem_* − *MR_rest_* − x*_3_* − x*_2_) and a time constant τ_la_. Here, the error signal is the difference between the total metabolic demand rate and the sum of the current aerobic metabolic rate and current lactic metabolic rate. Additionally, the response of the lactic system is dampened by the factor *K* times the rate of change of aerobic metabolic rate.

Furthermore, in Eq. (5), $\dot{x}_{1}$ is the alactic metabolic rate normed with the alactic energy capacity *E*_al,max_. Since the regulation of the alactic system acts directly on the alactic metabolic rate, the alactic system will instantly meet the metabolic demand that is not supplied by the lactic and aerobic systems. By norming with *E*_al,max_, the state variable *x*_1_ will have a feasible range of 0–1, where 0 equates to a fully recovered alactic system and 1 equates to completely depleted alactic system.

Finally, $\dot{x}_{4}$ is the rate of change of [mLa^−^] normed with the muscle lactate storage capacity *V*_m_, which gives the state variable *x*_4_ a feasible range of 0–1, where 0 equates to no accumulated muscle lactate (i.e., fully recovered lactic system) and 1 equates to maximum accumulation of muscle lactate (i.e., further utilization of the lactic system is limited). Muscle lactate is accumulated at a rate of *x*_2_/*V*_m_ and reduced by a maximum amplitude *A*_red_/*V*_m_ times the [mLa^−^] *x*_4_ times a demand factor *Z*_dem_, given by Eq. (6). The demand factor is equal to 1 when MR_dem_ is equal to MR_rest_ and decreases linearly to 0 when MR_dem_ equals the metabolic rate at the lactate threshold MR_lt_.

6 $$\left\{\begin{array}{l}{Z}_{\rm dem}=\frac{\text{M{R}}_{\rm lt}-\text{M{R}}_{\rm dem}}{\text{M{R}}_{\rm lt}-\text{M{R}}_{\rm rest}}\\ \text{M{R}}_{\rm dem}>\text{M{R}}_{\rm lt}\Rightarrow {Z}_{\rm dem}=0\\ \text{M{R}}_{\rm dem} < \text{M{R}}_{\rm rest}\Rightarrow {Z}_{\rm dem}=1\end{array}\right.$$

This means that an increased [mLa^−^] as well as a decreased MR_dem_ will give a faster rate of reduction in muscle lactate. Furthermore, to capture the behavior of an elevated steady state [bLa^−^] (and hence [mLa^−^]), the reduction of [mLa^−^] will cease from the lactate threshold (LT1). Since MR_ae_ may reach an elevated steady state above LT1 and since the lactic system in the proposed model describes the part of glycolysis resulting in lactate accumulation, this would cause the lactic glycolysis to cease. Hence, muscle lactate removal above LT1 would not result in a steady state [mLa^−^].

The given equations are supplemented by several conditions. Should the alactic metabolic rate *ẋ*_1_ fall below zero, only part of the energy diverted to the alactic recovery is converted into usable alactic energy due to the inevitable hysteresis cost of recovery. This is expressed in Eq. (7), where η_al_ is the efficiency of alactic recovery and thus the hysteresis cost is (1 −${\eta }_{\mathrm{al}}$)·$\dot{x}_{1}$.

7 $${\dot{x}}_{1}<0\Rightarrow {\dot{x}}_{1}={\eta }_{\mathrm{al}}\cdot {\dot{x}}_{1}$$

Recovery of the lactic system is linked to the reduction of accumulated muscle lactate given in Eq. (5). The anaerobic glycolysis itself is not allowed to fall below 0, as stated by Eq. (8).

8 $$\begin{array}{ll}\left\{\begin{array}{l}{x}_{2}=0\\ {\dot{x}}_{2}<0\end{array}\right.& \Rightarrow {\dot{x}}_{2}=0\end{array}$$

Finally, the MR_ae_ cannot exceed MR_ae,max_ (the energetic equivalent of $\dot{V}{\text{O}}_{{{\text{2max}}}}$), hence *x*_3_ is limited by MR_ae,max_ − MR_rest_, as given by Eq. (9).

9 $$\left\{\begin{array}{ll}\begin{array}{l}{x}_{3}=\text{M{R}}_{\rm ae,max}-\text{M{R}}_{\rm rest}\\ {\dot{x}}_{3}>0\end{array}& \Rightarrow {\dot{x}}_{3}=0\end{array}\right.$$

#### Energy demand

The remaining model equations define the components of metabolic demand. Adding the metabolic demands together, including MR_rest_, the total metabolic demand is given by Eq. (10).

10 $${\mathrm{MR}}_{\mathrm{dem}}={\mathrm{MR}}_{\mathrm{rest}}+{\mathrm{MR}}_{\mathrm{f}}+{\mathrm{MR}}_{\mathrm{ve}}+{\mathrm{MR}}_{\mathrm{acc}}$$

The fundamental metabolic demand rate MR_f_ is modelled by Eq. (11) as a linear function of the measured power output from the ergometer *u*_1_.

11 $$\left\{\begin{array}{l}\text{M{R}}_{\rm f}={A}_{\rm f}+{B}_{\rm f}\cdot {u}_{1}\\ {u}_{1}=0\Rightarrow \text{M{R}}_{\rm f}=0\end{array}\right.$$

*A*_f_, in theory, is the metabolic demand rate of unloaded cycling and *B*_f_ the increase in metabolic demand per unit increase in power output. Additionally, MR_f_ is set to 0 when the power output is 0, i.e., during passive rest. The metabolic demand due to ventilation is not included in MR_f_. Hence, MR_f_ is different from the often-used linear relationship between metabolic rate and power output that can be established at submaximal workloads.

The metabolic demand rate due to ventilation MR_ve_ has been shown to vary non-linearly with minute ventilation but with large inter-individual differences (Vella et al. 2006). Here, it is assumed that a single relationship between MR_ve_ and the measured minute ventilation *u*_2_ is valid for all measured *u*_2_ and that in theory *u*_2_ = 0 would result in MR_ve_ = 0. Hence, MR_ve_ is given by Eq. (12), where *A*_ve_ is the maximum metabolic demand rate due to ventilation, $\dot{V}_{{E{\text{,max}}}}$ is the maximum measured ventilation during P2, and *B*_*ve*_ is a distributing factor between a linear and a quadratic response. Both *u*_2_ and $\dot{V}_{{E{\text{,max}}}}$ are calculated as body temperature, pressure, water vapor saturated (BTPS).

12 $$\text{M{R}}_{\rm ve}={A}_{\rm ve}\left({B}_{\rm ve}\left(\frac{{u}_{2}}{{\dot{V}}_{\rm E,max}}\right)+\left(1-{B}_{\rm ve}\right)\left(\frac{{u}_{2}^{2}}{{\dot{V}}_{\rm E,max}}\right)\right)$$

The assumption that Eq. (12) is valid for all measured *u*_*2*_ is the reason metabolic demand due to ventilation is not included in MR_f_ and MR_rest_.

Metabolic demand associated with the accumulation of metabolites in the muscles MR_acc_ is given by Eq. (13). Though the accumulation of lactate itself may be the cause of a rise in metabolic demand due to lactate shuttling, the [mLa^−^] in this model is used as a proxy for the concentration of various accumulated metabolites that may cause additional metabolic demands (Gaesser and Brooks 1984). The metabolic demand associated with accumulated metabolites is given the same form as the metabolic rate from ventilation.

13 $$\text{M{R}}_{\rm acc}={A}_{\rm acc}\left({B}_{\rm acc}\cdot {x}_{4}+\left(1-{B}_{\rm acc}\right)\cdot {x}_{4}^{2}\right)$$

*A*_acc_ is the maximum amplitude of metabolic demand from accumulated metabolites and *B*_acc_ is the distributing factor between a linear and a quadratic response.

#### Model individualization through parameter estimation

In total, the described bioenergetic model includes 17 parameters that can be adjusted to individually adapt the model. Of these, 14 were individually estimated using the non-linear grey-box parameter estimation algorithm (*nlgreyest*) in the Systems Identification Toolbox in MATLAB (R2020b, Mathworks Inc., Natick, MA, United States), based on Lidar et al. (2021). This algorithm minimizes the mean squared error between measured and model data by iteratively adjusting the model parameters and solving the system of ordinary differential equations in the bioenergetic model (Eq. 4). To prevent feasible-range violation of the *x*_*1*_ and *x*_*2*_ state variables, a multiplicative penalty function was applied to the dependent variable (MR_ae_). If either of these state variables came close to its limit, MR_ae_ would rise rapidly, causing a higher error between measured and modeled data. To enforce a [mLa^−^] close to the maximum of 1 at exhaustion, [mLa^−^] was forced to exceed 0.85, 100 s prior to exhaustion during P2. A multi-start algorithm was implemented to increase the likelihood of global optima convergence, i.e., multiple initial values were set for selected parameters (see Table 2) and the parameter estimation algorithm was executed with all combinations of the initial values. The set of parameters that rendered the smallest mean squared error from the multi-start parameter estimation was considered the global optimal solution.

**Table 2:** Bioenergetic model parameters and constants with optimized individual values from the parameter estimation as mean ± SD (*n* = 14), initial values used in the parameter estimation, allowed ranges, and a brief description

Parameter	Optimized	Initial	Range	Description
${E}_{\mathrm{al},\mathrm{max}}$ (kW)	16.3 ± 1.7	21.0	–	Alactic energy capacity
${\tau }_{\mathrm{ae}}$ (s)	25.8 ± 1.4	25	[10; 100]	Time constant of aerobic system
${\mathrm{MR}}_{\mathrm{rest}}$ (W)	143 ± 27	135 ± 26^a^	[70; 220]	Metabolic rate at rest w/o ventilation
${\mathrm{MR}}_{\mathrm{lt}}$ (W)	952 ± 121	960 ± 103^b^	[70%; 130%]^c^	Metabolic rate at lactate threshold
${\mathrm{MR}}_{\mathrm{ae},\mathrm{max}}$ (W)	1649 ± 114	1674 ± 113^a^	[100%; 105%]^c^	Maximum aerobic metabolic rate
$\dot{V}_{E,\max }$ (L·min^−1^)	–	197 ± 12^a^	Constant	Maximum minute ventilation
${A}_{\mathrm{f}}$ (W)	158 ± 20	143, 158	[50; 250]	*Y*-intercept of fundamental work
${B}_{\mathrm{f}}$	2.96 ± 0.09	2.8	[2.2; 3.5]	Linear factor of fundamental work
${A}_{\mathrm{ve}}$ (W)	182 ± 23	8.8%^d^	[5%; 15%]^d^	Max metabolic rate due to ventilation
${B}_{\mathrm{ve}}$	0.93 ± 0.02	0.9	[0;1]	Linear-quadratic distribution factor of *MR*_*ve*_
${A}_{\mathrm{acc}}$ (W)	114 ± 20	3.8%^d^, 4.2%^d^	[0%; 15%]^d^	Max metabolic rate due to [mLa^−^]
${B}_{\mathrm{acc}}$	0.97 ± 0.08	1	[0; 1]	Linear-quadratic distribution factor of *MR*_*acc*_
${A}_{\mathrm{red}}$	0.48 ± 0.06%^e^	0.48%^e^, 0.53%^e^	[0.3%; 0.7%]^e^	Max amplitude of [mLa^−^] reduction
${\eta }_{\mathrm{al}}$	0.41 ± 0.01	0.40, 0.45	[0.4; 0.5]	Efficiency of alactic recovery
${V}_{\mathrm{m}}$ (kW L mol^−1^)	41.7 ± 2.1	38.8, 41.2	[50%; 150%]^c^	Lactate volume capacity of active muscles
${\tau }_{\mathrm{la}}$ (s)	12.87 ± 0.52	11.9, 13.1	[10; 15]	Time constant of lactic system
$K$	–	0.8	Constant	Dampening factor of lactic system

All combinations of the initial values were used in the parameter estimation, totaling 64 initial value combinations. Initial values reported as mean ± SD indicate that individual initial values based on P1a and/or P2 were used. *K* was set to 0.8 for all subjects based on preliminary parameter estimations

^a^Individual values taken from P2 measurements

^b^Individual values taken from P1a measurements

^c^Percentage of initial parameter value

^d^Percentage of ${\mathrm{MR}}_{\mathrm{ae},\mathrm{max}}$ initial value

^e^Percentage of *V*_m_ initial value

Three of the 17 parameters were treated separately. The maximum minute ventilation $\dot{V}_{{E{\text{,max}}}}$ was considered an individual constant with values set as the maximum measured $\dot{V}_{E}$ during P2 for each subject. The alactic system is the buffer of the bioenergetic system and, as such, it does not affect the behavior of the lactic or aerobic systems, nor metabolic demand. Therefore, the alactic capacity *E*_al,max_ was adjusted after the parameter estimation to the initial value times the maximum achieved value of *x*_1_ during P2. Finally, the smoothing factor *K* was set to a constant value of 0.8 since preliminary parameter estimations showed that lower and higher values resulted in unreasonably low and high contributions from the lactic system respectively.

To avoid infeasible parameter estimations, upper and lower limits were set for the 14 parameters in the parameter estimation. For MR_rest_, MR_lt_, and MR_ae,max_, the initial values (Table 2) were approximated from P2 measurements (except MR_lt_ from P1a). Since the approximation of MR_ae,max_ was the measured maximal 60 s mean during P2, it was considered a reasonable but underestimated approximation, and hence was limited to a maximum increase of 5% (no allowed decrease). Preliminary parameter estimations yielded unreasonably low and high values of MR_rest_, but with absolute limits preventing these extreme values there were only minor deviations from the initial values. MR_lt_ was limited to a variation between 70% and 130% of its initial value.

The aerobic time constant τ_ae_ was limited to a variation between 10 and 100 s (di Prampero 1981) with an initial estimate of 25 s (Artiga Gonzalez et al. 2019). The initial value of τ_la_ was established from Fig. 5 in Gastin (2001), in which energy demand is ~ 73 ml O_2_ equivalent·kg^−1^·min^−1^. If glycolysis were to continue toward this value without any contribution from the aerobic system, it would attain 63% after one time constant, which is about 46 ml O_2_ equivalent·kg^−1^·min^−1^. Sketching this hypothetical curve, the value of 46 ml O_2_ equivalent·kg^−1^·min^−1^ would be reached after approximately 12.5 s. Large variations from this approximation yielded unreasonable results in preliminary parameter estimations, and hence *τ*_la_ was limited to between 10 and 15 s.

The initial values of *A*_ve_ were set to 8.8% of the approximated MR_ae,max_ according to the mean oxygen cost of ventilation in the study by Vella et al. (2006). The allowed range was set to 5–15% of MR_ae,max_, where the lower limit was the lowest value according to Vella et al. (2006), but the upper limit was the approximate maximum cost of ventilation found by Aaron et al. (1992), since the highest value from Vella et al. (2006) stood out compared to similar studies. It was assumed that the imposed metabolic demand related to accumulated metabolites would be less than that related to ventilation. As such, initial values of *A*_acc_ were set to 3.8% or 4.2% of MR_ae,max_ (about half of *A*_ve_), but with the same limits as *A*_ve_, since no reasonable indications of its magnitude could be found in the literature. Initial values of *A*_f_, *B*_f_, *B*_ve_, *B*_acc_, *A*_red_, *η*_al_, and *V*_m_, were all chosen from preliminary parameter estimation results. The limits for *A*_c_ and *B*_c_ were applied to avoid unreasonable results, but even so, no values outside these ranges were obtained during the preliminary parameter estimations. The lower and upper limits of *B*_ve_ and *B*_acc_ were set to 0 and 1, respectively, since these parameters give the distribution between a linear and quadratic response in their respective equations. The limits of *A*_red_ and *V*_m_ were both set as percentages of *V*_m_. Since the values of these parameters were uncertain, *A*_red_ was allowed to vary more than ± 30% and *V*_m_ was allowed to vary ± 50% of their respective initial values.

All parameters’ initial values, estimated values, and allowed ranges are summarized in Table 2.

### Data collection

**Fig. 2:**
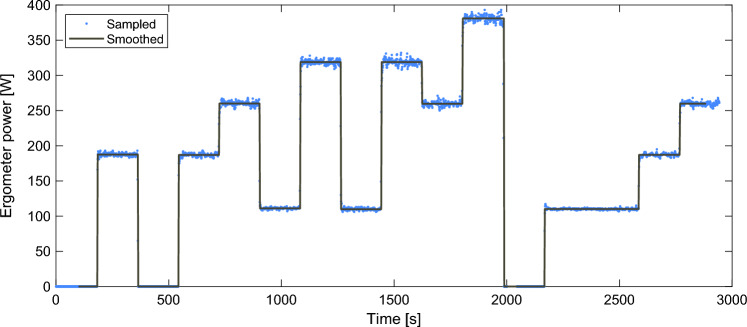
Sampled and smoothed ergometer power output for one representative subject for P2. The data was smoothed by setting the power output equal to the mean power output for each section. Additionally, the 100 first seconds and final 60 s were deleted to avoid smoothing error and reduce calculation times

All tests were performed on a belt-braked cycle ergometer (Monark LC7 TT Novo) with the power output and cadence sampled at 1 Hz. The power output was smoothed by setting the power output of each period to be equal to the mean power output for the same period. To reduce the number of data points and hence the calculation time for parameter estimation, the power output was resampled to 0.5 Hz using linear interpolation. An example of the measured and smoothed power output is shown in Fig. 2. All subjects used their own personal cycling shoes and were allowed to use their own pedals. Prior to the first protocol, all subjects adjusted saddle height and handlebar position (height and forward/backward distance) to their preference. These measurements were recorded and used for all subsequent test protocols.

Venous blood samples for analyses of [bLa^−^] were taken using 1-ml syringes from a 10 cm extension set connected to a catheter (Discofix® C; Vasofix® Safety; 1.1 × 25 mm, B. Braun Melsungen AG, Melsungen, Germany) in the cephalic vein or, in some cases, the mediana cubiti vein. Between the samples, the system was flushed with isotonic saline to avoid coagulation. Thus, each sampling started with discharging a volume greater than 2 ml before the actual sample was taken. The [bLa^−^] samples were analyzed using laboratory equipment (Biosen S-line; EKF-Diagnostic, Cardiff, UK) within 10 min of completion of P1b, P2, and P3 respectively.

**Fig. 3:**
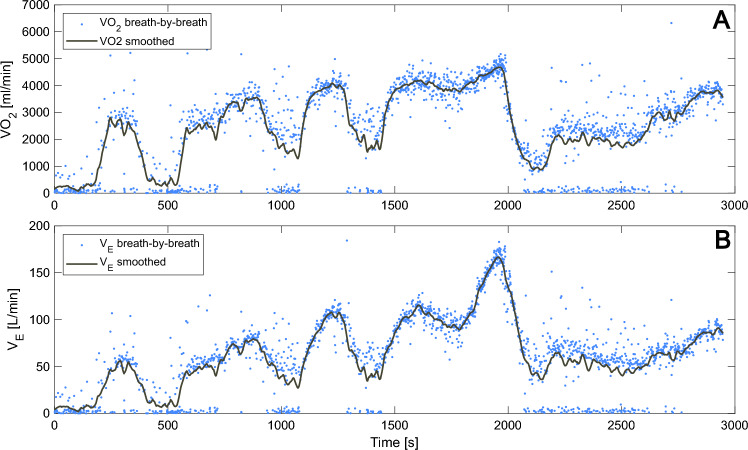
Sampled and smoothed breath-by-breath oxygen uptake (Panel **A**) and expiratory minute ventilation as STPD (Panel **B**) for one representative subject for P2. Data was smoothed using locally weighted linear regression with a 40-s time window and resampled to 0.5 Hz using linear interpolation

The subjects’ breath-by-breath expiratory minute ventilation and fractions of expired oxygen and carbon dioxide, were measured using an automated metabolic measurement system (Moxus Modular Metabolic System; AEI Technologies Inc., Pittsburg, USA) described in a previous paper (Ainegren et al. 2018). For the individual power output calculations, 60-s averages of the $\dot{V}{\text{O}}_{{2}}$ and RQ reported by the system were used. For the parameter estimation and model performance assessment, data from the metabolic measurement system was preprocessed in MATLAB. The inspiratory minute ventilation was calculated from the expiratory minute ventilation and fractions of inspired and expired nitrogen and expired oxygen and carbon dioxide using the Haldane transformation. The minute ventilations and gas fractions were smoothed using locally weighted linear regression (*smoothdata* with *lowess* method) with a 40-s time window to reduce the noise and obtain the underlying mean curve (Cleveland 1979). Gaps in the data during periods in which the participants removed the spirometry mouthpiece were filled using autoregressive modeling (*fillgaps*). From this smoothed data, $\dot{V}{\text{O}}_{{2}}$, carbon dioxide output, and RQ were calculated. Finally, MR_ae_ was calculated from $\dot{V}{\text{O}}_{{2}}$ and RQ according to Eq. (1). Expiratory minute ventilation as BTPS (*u*_1_) was used as an input for the calculation of MR_ve_, since it conveys actual expired air with current air temperature and density, but for all other calculations standard temperature, pressure, dry air (STPD) was used. To remove errors introduced by smoothing at the beginning and the end of the protocols, the first 100 s and last 60 s of the data was omitted and the data was resampled to 0.5 Hz using linear interpolation. This was also done to reduce the number of data points and hence the calculation time for parameter estimation. The first 100 s contained about half of the initial sitting rest period and the last 60 s contained a third of the concluding period (L2 for P2 and AR for P3). The breath-by-breath $\dot{V}{\text{O}}_{{2}}$ and expiratory minute ventilation as STPD ($\dot{V}_{{E{\text{,STPD}}}}$), as reported by the metabolic measurement system, is compared to the smoothed data for one subject in Fig. 3.

The individualized bioenergetic models were used for numerical simulation of P2 and P3. In those simulations *y* (MR_ae_), MR_la_, MR_al_, *x*_1_, MR_dem_, MR_f_, MR_ve_, MR_acc_, and *x*_4_ ([mLa^−^]) were calculated with measured power output and minute ventilation as BTPS as inputs.

### Statistical analysis

Data are presented as mean ± SD unless otherwise specified. As a measure of the overall model agreement with measurements, the root mean squared error (RMSE) in *W* between measured and modeled MR_ae_ was calculated with the time-resolved data (0.5 Hz) for each subject. Using the same data, the mean absolute percentage error (MAPE) was calculated with measurement data as reference and used as an overall measure of the model’s agreement with the measurements in relative terms.

The calculated mean ± SD of RMSE and MAPE are considered metrics of the validity, while SD of RMSE and MAPE alone are considered metrics of the reliability. Data from P2 is considered to show the validity and reliability reflecting the combined error of the model formulation, parameter estimation, data acquisition, and data preprocessing, while data from P3 also includes errors from day-to-day variation and protocol differences. The method of parameter estimation has been applied in several studies (Artiga Gonzalez et al. 2019; Lidar et al. 2021) and is believed to find close-to-optimal solutions. Also, the validity of the metabolic measurement system has been tested (Beltrami et al. 2014). Hence, data from P2 is believed to primarily to show validity and reliability of the model formulation and data preprocessing, and the difference between P2 and P3 is believed to primarily highlight the day-to-day variation and protocol specificity.

To further evaluate the model-to-measurement agreement, Bland–Altman plots (Bland and Altman 1999) were drawn with all data points from the time-resolved data (0.5 Hz) for P2 and P3 respectively. This identifies the mean difference ± 95% limits of agreement of MR_ae_.

Statistical parametric mapping (SPM) has been used for statistical inference in neuroimaging (Friston et al. 2007), but has also been shown applicable to one-dimensional data (Pataky 2010). In the case of an SPM *t*-test; residuals, variances, and finally *t*-statistics are calculated from a general linear model for each node in the data (Pataky 2010). Since one-dimensional data (e.g., time series) usually exhibit spatiotemporal smoothness, a corrected critical *t*-value is established from the geometry of the data and the estimated level of smoothness using random field theory (Pataky 2010, Appendix A). In this study, SPM was used to perform two-tailed paired *t*-tests on the measured and modeled MR_ae_ normalized with the individual parameter MR_ae,max_ for the entire time series for P2 (1379 nodes) and P3 (1079 nodes), respectively. Calculations were made with the open-source SPM 1D software (Pataky 2012) in MATLAB. An alpha level of < 0.05 was chosen with a null hypothesis of there being no difference between measured and modeled data. For the statistical inference, *p*-values are reported for each cluster of adjacent nodes where the *t*-statistics exceed the established critical *t*-value for P2 and P3, respectively. The results from the SPM analysis were also used to investigate if any specific parts of the intermittent protocols show signs of greater disagreement.

## Results

The RMSE of the individual MR_ae_ between measured and modeled data was 61.9 ± 7.9 W and the MAPE was 8.6 ± 1.5% for P2. For P3, the corresponding values were 79.2 ± 30.5 W and 10.6 ± 3.3%, respectively.

Results for the individualized parameters are shown in Table 2. Expressed as percentage of MR_ae,max_, *A*_ve_ was 11.0 ± 1.1% and *A*_acc_ was 6.9 ± 1.3%. In Table 3 the modeled overall metabolic energy contributions up to exhaustion from the three energy supply systems are shown for P2 and P3, respectively. Additionally, the maximum value of *x*_1_ during P3 was 1.12 ± 0.03 (by definition 1.0 ± 0.0 during P2) meaning 12% more than the estimated maximum alactic capacity was used during P3. Also, the maximum value of *x*_4_ during P3 was 1.21 ± 0.10 (0.99 ± 0.00 during P2) meaning on average 21% higher [mLa^−^] than the estimated maximum from P2 was achieved during P3.

**Table 3:** Modeled metabolic energy contributions up to exhaustion from the two intermittent test protocols (P2 and P3) respectively, reported as amount of energy (kJ) and as percentage of total metabolic energy output

Metabolic energy system	Energy contributions during P2		Energy contributions during P3	
	[kJ]	%	[kJ]	%
Alactic	30.4 ± 5.7	3.3 ± 0.4	41.9 ± 8.0	4.4 ± 0.6
Lactic	29.2 ± 2.5	3.1 ± 0.3	33.7 ± 3.7	3.6 ± 0.3
Aerobic	873 ± 73	93.6 ± 0.5	872 ± 78	92.0 ± 0.8
Anaerobic	59.6 ± 7.3	6.4 ± 0.5	75.6 ± 10.4	8.0 ± 0.8

The first 100 s of passive rest in each protocol was omitted (see Data Collection)

**Fig. 4:**
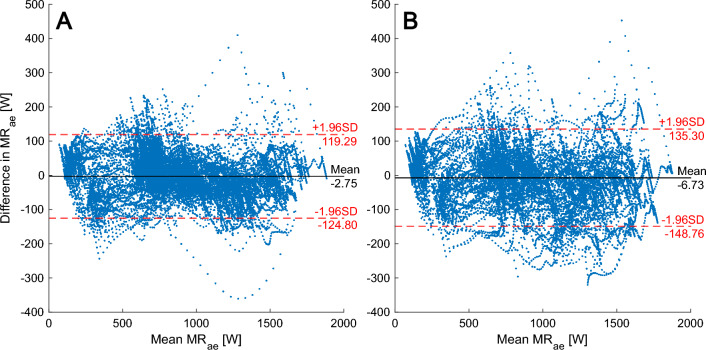
Bland–Altman plot showing the mean of and difference between measured and modeled MR_ae_ for all data samples from all subjects during P2 (Panel **A**) and P3 (Panel **B**) including the model-to-measurement mean difference and 95% confidence intervals. 94.6% of the data points reside inside the confidence interval for P2 and 94.0% for P3

The differences between measured and modeled MR_ae_ in relation to mean MR_ae_ are shown with a Bland–Altman plot for P2 in Fig. 4A and for P3 in Fig. 4B. The mean difference shows a small underprediction by the model in P2 and an increased underprediction in P3. The confidence intervals show there are greater overall differences in P3 compared to P2. There is no obvious negative or positive trend in the data across the range of mean MR_ae_.

**Fig. 5:**
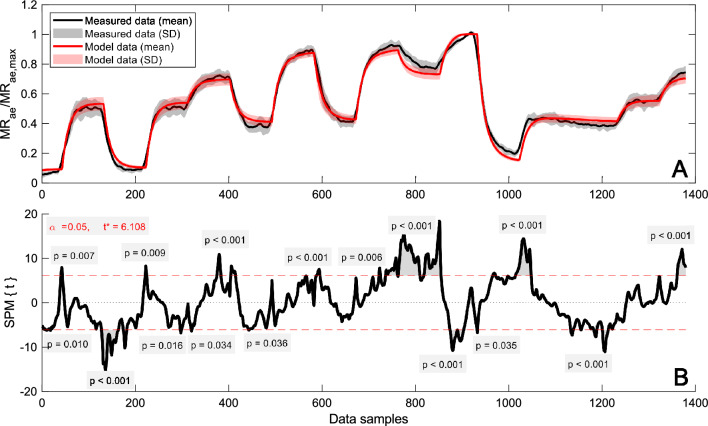
Time resolved mean and SD (Panel **A**) and statistical parametric mapping from the two-tailed paired *t*-test (Panel **B**) of measured and modeled individual MR_ae_ normalized with MR_ae,max_ for P2 (*n* = 11). Results from three subjects are omitted due to deviations in duration during one or more periods (same subjects as in Fig. 6). Data for the period leading to volitional exhaustion has been stretched/squeezed to allow comparison across the subjects. The dashed lines show the critical *t*-value, indicating the limit for significant differences (*p* < 0.05) between measured and modeled data

**Fig. 6:**
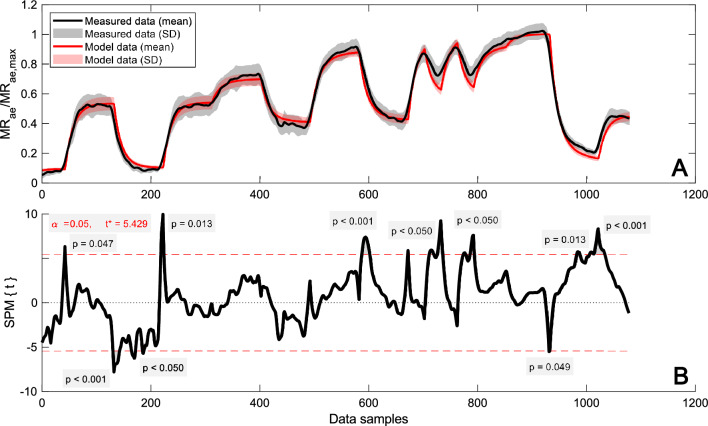
Time resolved mean and SD (Panel **A**) and statistical parametric mapping from the two-tailed paired *t*-test (Panel **B**) of measured and modeled individual MR_ae_ normalized with MR_ae,max_ for P3 (*n* = 11). Results from three subjects are omitted due to deviations in duration during one or more periods (same subjects as in Fig. 5). Data for the period leading to volitional exhaustion has been stretched/squeezed to allow comparison across the subjects. The dashed lines show the critical *t*-value, indicating the limit for significant differences (*p* < 0.05) between measured and modeled data

Visually, Fig. 5A shows overall excellent agreement in the time resolved MR_ae_/MR_ae,max_ averaged across the subjects (*n* = 11) from measurement and model data, respectively. Still, significant differences between the model and measurements occur at several different stages during P2 (Fig. 5B). Figure 6A shows the corresponding results for P3, also with good overall agreement. The number of clusters with a *t*-value above the critical *t*-value are of the same magnitude as in P2 but less prominent (Fig. 6B), which is likely explained by the larger SD of measurements in P3 (Fig. 6A).

**Fig. 7:**
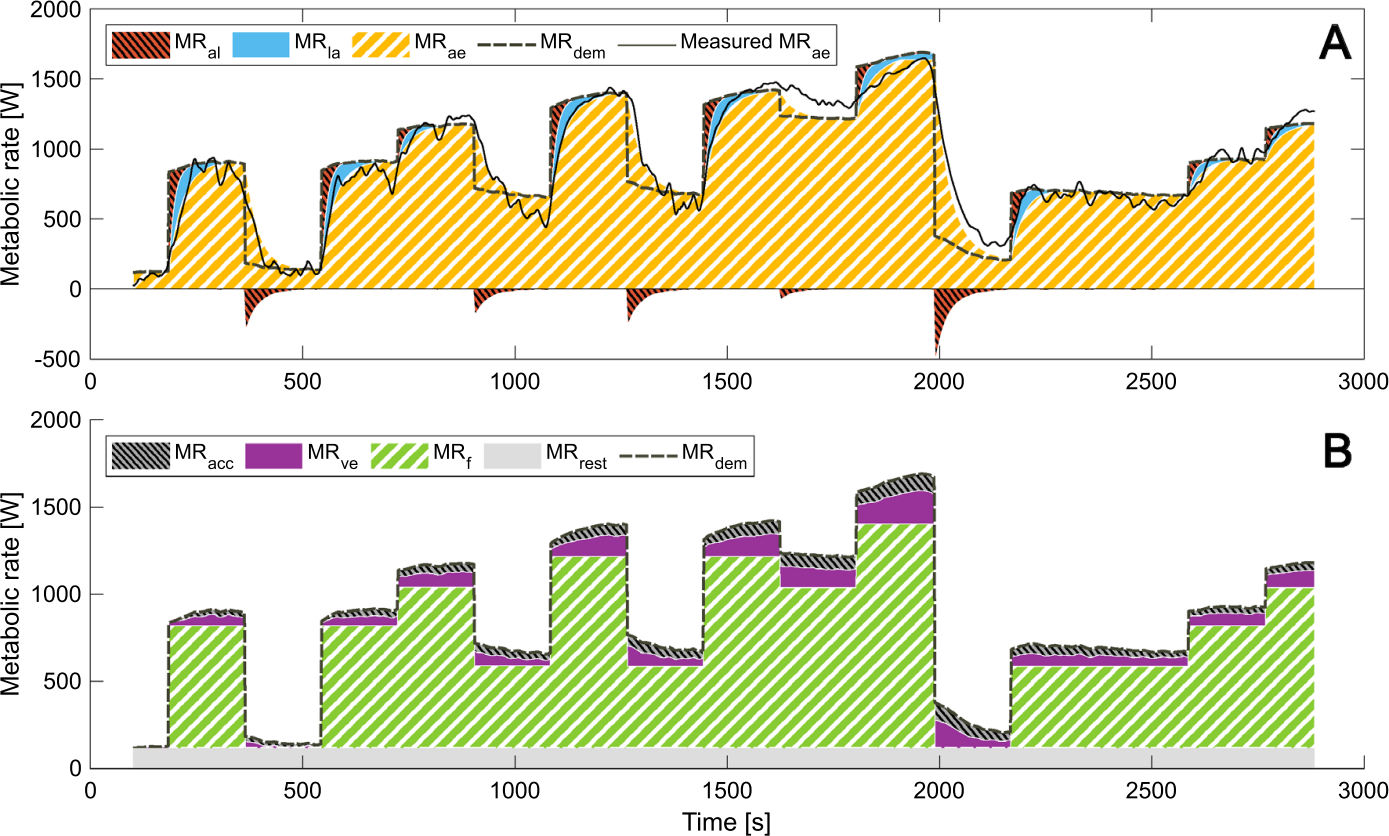
Metabolic supply rates (Panel **A**) and metabolic demand rates (Panel **B**) for P2 in a representative subject (S8). MR_al_ is alactic metabolic rate, MR_la_ lactic metabolic rate, MR_ae_ modeled aerobic metabolic rate, MR_dem_ total metabolic demand rate and Measured MR_ae_ is the smoothed measured aerobic metabolic rate. MR_acc_ is metabolic demand rate due to accumulated metabolites, MR_ve_ metabolic demand rate due to ventilation, MR_f_ metabolic demand rate due to the fundamental work and MR_rest_ the metabolic demand rate at rest. In Panel **A** MR_rest_ is included in MR_ae_

**Fig. 8:**
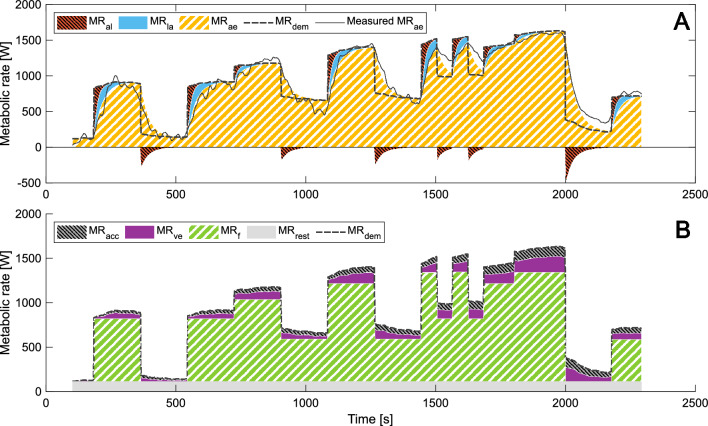
Metabolic supply rates (Panel **A**) and metabolic demand rates (Panel **B**) for P3 in a representative subject (S8). MR_al_ is alactic metabolic rate, MR_la_ lactic metabolic rate, MR_ae_ modeled aerobic metabolic rate, MR_dem_ total metabolic demand rate and Measured MR_ae_ is the smoothed measured aerobic metabolic rate. MR_acc_ is metabolic demand rate due to accumulated metabolites, MR_ve_ metabolic demand rate due to ventilation, MR_f_ metabolic demand rate due to the fundamental work and MR_rest_ the metabolic demand rate at rest. In Panel **A** MR_rest_ is included in MR_ae_

Figures 7A and 8A show the modeled energy systems contributions for one representative subject during P2 and P3, respectively. The contributions from different terms to MR_dem_ are shown in Figs. 7B and 8B during P2 and P3 respectively. There is an obvious difference between MR_dem_ during the initial passive rest periods and the passive rest following exhaustion caused by the differences in MR_ve_ and MR_acc_.

## Discussion

### Model validity and reliability

The proposed bioenergetic model shows good overall agreement with the measurements of MR_ae_. The estimated values of MR_ae,max_, MR_lt_, and MR_rest_ are similar to their estimated values from measurements. The estimated values of τ_ae_ agree with earlier findings (Artiga Gonzalez et al. 2019), as do the estimated values of MR_ve_ (Vella et al. 2006). The anaerobic energy contributions were on average 59.6 and 75.6 kJ during P2 and P3 respectively. The anaerobic energy contribution for one well-trained cyclist during a mass start cycling race was estimated to about 35 kJ (Skiba et al. 2012, Fig. 4). On the other hand, the mean anaerobic capacity is 93.2 kJ when estimated from the body masses for the subject in this study according to the findings of Andersson et al. (2022) regarding 3 min cycling time trials. Since the glycolysis is affected by the rate of accumulation and reduction of muscle metabolites (Stanley and Connett 1991), the exercise intensity and protocol will likely affect the lactic energy system contribution. Hence, it is problematic to define a general anaerobic capacity. The results in this study at least do not stand out as unreasonable.

The combined validity of the model formulation, parameter estimation, data acquisition, and data preprocessing (MAPE 8.6 ± 1.5% when fitted to P2 data) is not as good as the model proposed by Artiga Gonzalez et al. (2019), which, when fitted to an intermittent protocol, showed an MAPE of 3.4 ± 1.0%. However, applicability of the proposed model to a similar protocol, assessed as the difference in MAPE between P2 and P3, is seemingly better. MAPE was 10.6 ± 3.3% when the model fitted to P2 data was applied to P3; the model proposed by Artiga Gonzalez et al. (2019) showed MAPE of 10.8 ± 4.7% when the fitted model was applied to a race-like protocol. It should be noted that the protocols in this study are more similar, which is likely favorable for the proposed model. On the other hand, the smoothing applied to the data by Artiga Gonzalez et al. (2019) is likely favorable to their model in comparison to the smoothing applied in this study (this is further discussed below).

From the measured data in this study, it was visually obvious (not visible in this article) that three subjects exhibited larger systematic differences between P2 and P3. Quantifying the day-to-day variation, the RMSE between P2 and P3 of the last 60 s mean of the first 6 (submaximal) periods of each protocol was 46.5 ± 32.1 W for the whole group (*n* = 14), but 31.3 ± 7.9 W with these three subjects omitted (*n* = 11). The mean difference of the first 6 periods for these three subjects was 73.4, -70.9, and 101.7 W, respectively. With these subjects omitted, the overall RMSE of P3 was 65.2 ± 10.2 W (*n* = 11), compared to 79.2 ± 30.5 W for the whole group (*n* = 14). This may suggest greater potential for the proposed model as the RMSE of P3 and P2 are close (*n* = 11). However, mostly it emphasizes the limitations of predicting endurance performance with numerical modeling, with day-to-day variations having a greater influence than the relative accuracy of any proposed model.

### Performance quantification and comparison

Regarding RMSE and MAPE as metrics of model performance, it should be noted that the errors in both cases are calculated as the difference between the model predictions and measurements for each specific time sample. A minor misalignment in time between the results may give a comparatively large error, while the amplitude and shape of the curve could be perfectly matched. The implications of this will be greatest during transitions, when MR_ae_ is rapidly changing. This issue will also be present in the Bland–Altman plots, as these too are applied to matched time samples. The equivalent mean squared error measure is used when fitting the model parameters, so these problems should be minimized. Nevertheless, this obscures the comparability of results and argues for an alternative quantification of curve agreement. One possible suggestion could be the mean of the closest distance from each respective data sample from the evaluated time series to the entire reference time series.

An additional issue with quantifying the model performance is the smoothing of measured data, which will have greatest effect in sharp transitions. In this study specifically, a rapid rise or fall of MR_ae_ with a sudden change in demand will be smoothed to a somewhat more gradual change that starts earlier. Since the model is formulated to react to changes in demand, this will undoubtedly result in an error right before every major transition. A more gradual approach toward steady state is applied to the end of the transitions and, as such, these will not be as heavily affected. This phenomenon will directly affect the comparison of results between this study and the work of Artiga Gonzalez et al. (2019), since a greater degree of smoothing was applied to the dependent variable in the latter case. In addition, Artiga Gonzalez et al. (2019) smoothed the ergometer power output used as the model input (independent variable), which applied the above-mentioned smoothing effect in the transitions. Both these processes potentially reduce the error between the model and measurements, thus introducing some false validity. On the contrary, the power output in this study was smoothed in a fashion that retained the sharp transitions.

### Physiological aspects

The proposed model provides possible descriptions of both the underlying mechanisms of the metabolic demand and the underlying control mechanisms of each of the energy supply systems. Unlike no previous model that we know of, the proposed model introduce feedback mechanisms from the metabolic energy supply system to the corresponding demand as an alternative mechanism for the $\dot{V}{\text{O}}_{{2}}$ slow component and the fatigue-induced gross efficiency degradation observed in cycling (Noordhof et al. 2015). Even so, the model must be considered a crude simplification of the real underlying mechanisms. Several other possible model variations and combinations were tested during preliminary parameter estimations. Reduced muscle efficiency was implemented as a factor proportional to [mLa^−^] and multiplied by MR_f_. Even though such an effect has been reported (Hopker et al. 2017), this resulted in no improvement in RMSE but gave a somewhat random relative distribution between MR_ve_, MR_acc_, and the metabolic demand due to reduced muscle efficiency. This could be a sign of the system of equations being underdetermined in relation to input data.

The model proposed in this study uses Watts as the standard unit for metabolic rate, and, as such, measured oxygen uptake is recalculated into aerobic metabolic rate before model fitting or comparison. As a first note, the recalculation takes RQ into account, but this is not treated separately by the model, which could lead to small errors, especially at lower exertions and/or at the beginning of an exercise. No ramifications of this problem have been tested. Another related issue concerns including part of the glycolysis in the aerobic system i.e., the part of the glycolysis that produces the right amount of pyruvate to fuel mitochondrial respiration without increasing the concentration of accumulated lactate. In the model, this is included as a constant fraction of the aerobic system, but the level of glycolysis likely varies. Specifically, glycolysis is likely reduced in favor of mitochondrial respiration using the accumulated lactate as fuel during recovery following intense exercise (Gaesser and Brooks 1984). Aerobic energy output per oxygen input without glycolysis should be less than 10% lower than with full glycolysis, calculated using adenosine triphosphate output from the involved processes. This effect would be highest during passive or active rest e.g., when MR_ae_ is less than 300 W resulting in an error less than 30 W. Preliminary testing was performed with a model compensating for this effect using an additional linearly dependent cost on MR_dem_ below the maximum lactate steady state and with a maximum amplitude of 10% of MR_ae_. However, as this effect is limited, it was removed from the proposed model for the purpose of simplicity.

A two-compartment lactate accumulation model was tested initially, where lactate accumulated in the muscles could transfer into another compartment defined as blood and other tissue. Reduction of lactate was also possible in this second compartment to reflect the fact that the cardio-pulmonary system favors lactate as fuel when available (Gaesser and Brooks 1984). However, this introduced an additional ordinary differential equation that needed to be solved numerically as well as several additional parameters, again leading to an underdetermined system in relation to input data in which several significantly divergent parameter value combinations could yield similarly small values of RMSE. In addition, the division of glycolysis between the lactic and aerobic systems, in combination with a two-compartment lactate system, either fails to establish a steady state lactate concentration or calls for a pure curve fitting controlling mechanism rather than a physiologically plausible one. The two-compartment lactate accumulation model made it possible to include an additional metabolic rate related to the transfer of lactate. This aspect is not included in the proposed model, which has only one compartment for lactate accumulation and does not distinguish between lactate transfer and lactate reduction through oxidation in the muscles.

As P2 and P3 are intermittent protocols of varying power output, it is uncertain if and how the slow aerobic component will be visible. The most characteristic deviation from the typical exponential behavior of MR_ae_ with a time constant around 25 s, can be seen in the example data for P2 in Fig. 7A. At the end of period 9, when the power output level drops, and during the following increase up to exhaustion, MR_ae_ more closely resembles a linear decrease and increase. It is also visible in the mean curves in Fig. 5A, as the same trend was seen for several subjects. We interpret this as being related to the slow component. It is also clear that the proposed model does not capture this behavior well. This behavior is also seen in P3, starting from the end of the first 1-min period and at least until the start of the last period leading up to exhaustion. The underprediction of MR_ae_ during this part of P3 leads to an overprediction of MR_la_, which partly explains why both *x*_*1*_ and *x*_*4*_ ([mLa^−^]) increases above 1 in P3 for all subjects. Especially during the 1-min periods of lower intensity more pronounced recovery than modeled may occur.

The behavior of the slow component has previously been captured by applying a second exponential function with another, significantly larger, time constant and a time delay (Özyener et al. 2001). However, the concept of a time delay only really works with protocols characterized by a single step response. With an intermittent protocol, several questions arise. For instance, at what exercise intensity should the time delay countdown start, and should the time delay restart after a low intensity period? Nevertheless, there are still underlying control mechanisms not captured with sufficient accuracy by the proposed model. Instead of introducing a second exponential, testing was conducted in which the time constant of the primary exponential τ_ae_ was increased exponentially (with varying degrees of power) as [mLa^−^] increased. This unfortunately rendered the model too unstable to provide reasonable solutions. However, exhaustion might be the result of metabolic demand starting to rise in an unstable manner. Thus, the idea of an exertion-dependent time constant might be worth investigating further.

## Conclusions

In summary, the present study proposes a new bioenergetic model which attempts to describe the dynamic behavior of the metabolic alactic, lactic and aerobic systems as well as contributions to the metabolic demand rates relating to both the fundamental ergometer cycling work, ventilation, and accumulation of muscle metabolites. A method of adapting the proposed model to reflect the bioenergetics of a specific athlete through grey-box parameter estimation is shown to be satisfactory with 14 estimated parameters. The validation of the model shows excellent overall agreement between measured and modeled aerobic metabolic rate during intermittent cycling by well-trained male cyclist. However, SD is 38.5% of the mean RMSE for P3, indicating not as good reliability. In part this is explained by day-to-day variation, but the model fails in the capturing the full details of the slow component and recovery periods, which likely makes it sensitive to protocol differences between parameter estimation and application. This argues for further development and validation to establish the full potential of the model.

## Data Availability

Raw data collected for this study are not publicly available to preserve individuals’ privacy under the European General Data Protection Regulation.

## List of symbols

*η*_al_: Efficiency of alactic recovery
*τ*_ae_: Time constant of aerobic system
*τ*_l__a_: Time constant of lactic system
*A*_acc_: Maximum metabolic demand rate due to accumulated metabolites
*A*_f_: Y-intercept of fundamental metabolic demand rate
AR: Active rest
*A*_red_: Maximum reduction of muscle lactate
*A*_ve_: Maximum metabolic demand rate due to ventilation
*B*_acc_: Linear-quadratic distributing factor
*B*_f_: Linear factor of fundamental metabolic demand rate
[bLa^−^]: Blood lactate concentration
*B*_ve_: Linear-quadratic distributing factor
BTPS: Body temperature, pressure, water saturated
*E*_al,max_: Alactic energy capacity
*K*: Lactic system dampening factor
L1, L2, L3: Power output levels in the intermittent protocols
L4.2: Power output level in intermittent protocol test day 2
L4.3: Power output level in intermittent protocol test day 3
LT1: Lactate threshold
[mLa^−^]: Representation of active muscle lactate concentration
MAPE: Mean absolute percentage error
MR_acc_: Metabolic demand rate due to accumulated metabolites
MR_ae_: Aerobic metabolic rate
MR_ae,max_: Maximum aerobic metabolic rate
MR_al_: Alactic metabolic rate
MR_dem_: Total metabolic demand rate
MR_f_: Fundamental metabolic demand rate
MR_la_: Lactic metabolic rate
MR_lt_: Metabolic rate at lactate threshold
MR_rest_: Metabolic rate at rest
MR_sup_: Total metabolic supply rate
MR_ve_: Metabolic demand rate from ventilation
*P*_LT1_: Power output at lactate threshold
*P*_max_: Maximum power output
*P*_OBLA_: Power output at onset of blood lactate accumulation
*P*_VO2,peak_: Power output at peak oxygen uptake
P1a: Submaximal incremental protocol
P1b: Maximal incremental protocol
P2: Intermittent protocol test day 2
P3: Intermittent protocol test day 3
PR: Passive rest
RMSE: Root mean square error
RQ: Respiratory quotient
SPM: Statistical parametric mapping
STPD: Standard temperature, pressure, dry air
*u*_1_: Ergometer power output
*u*_2_: Expiratory minute ventilation as BTPS
$\dot{V}_{E,\max }$: Maximum expiratory minute ventilation
$\dot{V}_{{E{\text{,STPD}}}}$: Expiratory minute ventilation as STPD
*V*_m_: Muscle lactate accumulation capacity
$$\dot{V}{\text{O}}_{{2}}$$: Oxygen uptake
$\dot{V}{\text{O}}_{{2,{\text{peak}}}}$: Peak oxygen uptake
$\dot{x}_{1}$ = MR_al_: Alactic metabolic rate
*x*_2_ = MR_la_: Lactic metabolic rate
*x*_3_: Dynamic component of the aerobic metabolic rate
*x*_4_: Accumulated muscle lactate
*y* = MR_ae_: Aerobic metabolic rate
*Z*_dem_: Demand factor

## References

[CR1] Aaron EA, Seow KC, Johnson BD, Dempsey JA (1992). Oxygen cost of exercise hyperpnea: implications for performance. J Appl Physiol.

[CR2] Ainegren M, Jensen K, Rosdahl H (2018). Breathing resistance in automated metabolic systems is high in comparison with the Douglas Bag method and previous recommendations. Proc Inst Mech Eng Part P J Sports Eng Technol.

[CR3] Andersson EP, Bachl P, Schmuttermair A, Staunton CA, Stöggl TL (2022). Anaerobic work capacity in cycling: the effect of computational method. Eur J Appl Physiol.

[CR4] Artiga Gonzalez A, Bertschinger R, Brosda F, Dahmen T, Thumm P, Saupe D (2019). Kinetic analysis of oxygen dynamics under a variable work rate. Hum Mov Sci.

[CR5] Beltrami FG, Froyd C, Mamen A, Noakes TD (2014). The validity of the Moxus modular metabolic system during incremental exercise tests: impacts on detection of small changes in oxygen consumption. Eur J Appl Physiol.

[CR6] Bland JM, Altman DG (1999). Measuring agreement in method comparison studies. Stat Methods Med Res.

[CR7] Cleveland WS (1979). Robust locally weighted regression and smoothing scatterplots. J Am Stat Assoc.

[CR8] di Prampero PE (1981). Energetics of muscular exercise. Rev Physiol Biochem Pharmacol.

[CR9] Friston K, Ashburner J, Kiebel S, Nichols T, Penny W (2007). Statistical parametric mapping: the analysis of functional brain images.

[CR10] Gaesser GA, Brooks GA (1984). Metabolic bases of excess post-exercise oxygen consumption: a review. Med Sci Sports Exerc.

[CR11] Gastin PB (2001). Energy system interaction and relative contribution during maximal exercise. Sports Med.

[CR12] Gløersen Ø, Gilgien M, Dysthe DK, Malthe-Sørenssen A, Losnegard T (2020). Oxygen demand, uptake, and deficits in elite cross-country skiers during a 15-km race. Med Sci Sports Exerc.

[CR13] Hopker JG, O’Grady C, Pageaux B (2017). Prolonged constant load cycling exercise is associated with reduced gross efficiency and increased muscle oxygen uptake. Scand J Med Sci Sports.

[CR14] Lidar J, Andersson EP, Sundström D (2021). Validity and reliability of hydraulic-analogy bioenergetic models in sprint roller skiing. Front Physiol.

[CR15] Margaria R (1976). Biomechanics and energetics of muscular exercise.

[CR16] McArdle WD, Katch FI, Katch VL (2009). Exercise physiology: nutrition, energy, and human performance.

[CR17] McCann DJ, Molé PA, Caton JR (1995). Phosphocreatine kinetics in humans during exercise and recovery. Med Sci Sports Exerc.

[CR18] Morton RH (1986). A three component model of human bioenergetics. J Math Biol.

[CR19] Morton RH (1990). Modelling human power and endurance. J Math Biol.

[CR20] Morton RH, Billat LV (2004). The critical power model for intermittent exercise. Eur J Appl Physiol.

[CR21] Moxnes JF, Sandbakk Ø, Hausken K (2014). Using the power balance model to simulate cross-country skiing on varying terrain. Open Access J Sports Med.

[CR22] Noordhof DA, Mulder RC, Malterer KR, Foster C, de Koning JJ (2015). The decline in gross efficiency in relation to cycling time-trial length. Int J Sports Physiol Perform.

[CR23] Özyener F, Rossiter HB, Ward SA, Whipp BJ (2001). Influence of exercise intensity on the on- and off-transient kinetics of pulmonary oxygen uptake in humans. J Physiol.

[CR24] Pataky TC (2010). Generalized n-dimensional biomechanical field analysis using statistical parametric mapping. J Biomech.

[CR25] Pataky TC (2012). One-dimensional statistical parametric mapping in Python. Comput Methods Biomech Biomed Eng.

[CR26] Poole DC, Jones AM (2012). Oxygen uptake kinetics. Comprehensive Physiol.

[CR27] Skiba PF, Chidnok W, Vanhatalo A, Jones AM (2012). Modeling the expenditure and reconstitution of work capacity above critical power. Med Sci Sports Exerc.

[CR28] Skiba PF, Fulford J, Clarke DC, Vanhatalo A, Jones AM (2015). Intramuscular determinants of the ability to recover work capacity above critical power. Eur J Appl Physiol.

[CR29] Stanley WC, Connett RJ (1991). Regulation of muscle carbohydrate metabolism during exercise. FASEB J.

[CR30] Stirling JR, Zakynthinaki MS, Saltin B (2005). A model of oxygen uptake kinetics in response to exercise: including a means of calculating oxygen demand/deficit/debt. Bull Math Biol.

[CR31] Sundström D (2016). On a bioenergetic four-compartment model for human exercise. Sports Eng.

[CR32] Vella CA, Marks D, Robergs RA (2006). Oxygen cost of ventilation during incremental exercise to VO_2_ max. Respirology.

